# Intratumoral Tumor Infiltrating Lymphocytes (TILs) are Associated With Cell Proliferation and Better Survival But Not Always With Chemotherapy Response in Breast Cancer

**DOI:** 10.1097/SLA.0000000000005954

**Published:** 2023-06-15

**Authors:** Rongrong Wu, Masanori Oshi, Mariko Asaoka, Li Yan, Matthew G.K. Benesch, Thaer Khoury, Masayuki Nagahashi, Yasuo Miyoshi, Itaru Endo, Takashi Ishikawa, Kazuaki Takabe

**Affiliations:** * Department of Surgical Oncology, Roswell Park Comprehensive Cancer Center, Buffalo, NY; † Department of Breast Surgery and Oncology, Tokyo Medical University, Tokyo, Japan; ‡ Department of Gastroenterological Surgery, Yokohama City University Graduate School of Medicine, Yokohama, Kanagawa, Japan; § Department of Biostatistics & Bioinformatics, Roswell Park Comprehensive Cancer Center, Buffalo, NY; ∥ Department of Pathology & Laboratory Medicine, Roswell Park Comprehensive Cancer Center, Buffalo, NY; ¶ Division of Breast and Endocrine Surgery, Department of Surgery, Hyogo Medical University School of Medicine, Nishinomiya, Hyogo, Japan; # Department of Surgery, Jacobs School of Medicine and Biomedical Sciences, State University of New York, Buffalo, NY; ** Department of Surgery, Niigata University Graduate School of Medical and Dental Sciences, Niigata, Japan; †† Department of Breast Surgery, Fukushima Medical University, Fukushima, Japan

**Keywords:** bioinformatics, neoadjuvant chemotherapy, subtype, survival, tumor microenvironment

## Abstract

**Objective:**

To investigate the clinical relevance of intratumoral tumor infiltrating lymphocytes (TILs) in breast cancer as measured by computational deconvolution of bulk tumor transcriptomes.

**Summary Background Data:**

Commonly assessed TILs, located in tumor stroma without direct contact with cancer cells (stromal TILs), correlate with breast cancer treatment response and survival. The clinical relevance of intratumoral TILs has been less studied partly due to their rarity; however, they may have nonnegligible effects given their direct contact with cancer cells.

**Methods:**

In all, 5870 breast cancer patients from TCGA, METABRIC, GSE96058, GSE25066, GSE163882, GSE123845, and GSE20271 cohorts were analyzed and validated.

**Results:**

The intratumoral TIL score was established by the sum of all types of lymphocytes using the xCell algorithm. This score was the highest in triple-negative breast cancer (TNBC) and the lowest in the ER-positive/HER2-negative subtype. It correlated with cytolytic activity and infiltrations of dendritic cells, macrophages, and monocytes, and uniformly enriched immune-related gene sets regardless of subtype. Intratumoral TIL-high tumors correlated with higher mutation rates and significant cell proliferation on biological, pathological, and molecular analyses only in the ER-positive/HER2-negative subtype. It was significantly associated with pathological complete response after anthracycline- and taxane-based neoadjuvant chemotherapy in about half of the cohorts, regardless of the subtype. Intratumoral TIL-high tumors correlated with better overall survival in HER2-positive and TNBC subtypes consistently in 3 cohorts.

**Conclusions:**

Intratumoral TILs estimated by transcriptome computation were associated with increased immune response and cell proliferation in ER-positive/HER2-negative and better survival in HER2-positive and TNBC subtypes, but not always with pathological complete response after neoadjuvant chemotherapy.

Cancer aggressiveness is determined not only by cancer cell phenotypes but also by the interaction they have with the host tumor microenvironment, including immune cells. Tumor infiltrating lymphocytes (TILs) have been repeatedly reported to be associated with breast cancer progression, treatment response, and survival outcomes in both experimental and clinical settings.^1,2^ However, TILs assessed in these studies were located in the tumor stroma between the clusters of cancer cells that do not directly interact with cancer cells (stromal TILs). The International TILs Working Group recommends measuring stromal TILs by pathological assessment because it is reproducible with minimal interobserver variability.^3^

Intratumoral TILs are the lymphocytes inside the bulk tumor that have direct contact with the cancer cells without stromal cell intervention.^3^ Although intratumoral TILs count strongly correlates with stromal TILs,^1,4^ it has been postulated that intratumoral TILs may be biologically more relevant than stromal TILs given their closer proximity to cancer cells.^3,5^ Studies that have evaluated stromal and intratumoral TILs separately have reported that intratumoral TILs were significantly associated with increased pathological complete response (pCR) rates after neoadjuvant chemotherapy.^1,5^ Nevertheless, investigations on intratumoral TILs are limited, partly due to the relatively low frequency of lymphocyte infiltration within the tumor compared with the stromal region, posing significant challenges in conducting qualitative and quantitative pathological assessments. The FinHER study evaluated both stromal and intratumoral TILs, but the data on intratumoral TILs were abandoned on final analysis due to the small number of lymphocytes that could be assessed on specimen sections.^6^ Caution is also needed when assessing intratumoral TILs on Hematoxylin and Eosin-stained sections because lymphocytes are mobile in living tissue, and therefore sections may not accurately represent the entire TIL population within a bulk tumor.^5^

To investigate the clinical relevance of intratumoral TILs, we estimated the fraction of TILs by transcriptomic scoring of bulk tumors. We analyzed publicly available cohorts, including The Cancer Genome Atlas (TCGA), which contains tumors that are composed of at least 60% to 80% tumor nuclei and less than 20% to 30% necrotic tissue. Therefore, we defined the TILs in the samples to be intratumoral TILs. Infiltration of 19 lymphocyte cell types was quantified using the xCell algorithm on the gene expressions within bulk tumors and their sum-defined intratumoral TIL quantity. Our group has conducted several bioinformatic analyses on robust numbers of patient cohorts and reported the association of breast cancer progression with the tumor microenvironment.^7–11^ The methodology used in this current study is similar to the ones we previously employed to report the clinical relevance of intratumoral CD8^+^ T-cells,^12^ intratumoral plasmacytoid dendritic cells,^13^ and regulatory T-cells.^14^ This approach is advantageous in that it allows objective determination of the TILs even when the number of cells is very small.

Given that intratumoral TILs have direct cell-to-cell contact with cancer cells, we hypothesized that their quantity has a strong correlation with breast cancer biology, namely cancer aggressiveness, and patient outcomes, including survival and response to neoadjuvant chemotherapy. Bioinformatic analyses were conducted on transcriptomic data in breast cancer to test our hypothesis, specifically by breast cancer subtypes.

## METHODS

### Data Retrieval and Processing

The data acquisition for the primary breast cancer cohorts included TCGA (*n*=1077),^15^ METABRIC (*n*=1094),^16^ GSE96058 (*n*=3069),^17^ GSE25066 (*n*=508),^18^ GSE163882 (*n*=222),^19^ GSE123845 (*n*=227),^20^ and GSE20271 (n=176).^21^ Processed data for TCGA and METABRIC cohorts were obtained from the cBioPortal for Cancer Genomics (https://www.cbioportal.org/).^22^ All TCGA samples were reviewed by pathologists at the time of collection to ensure they met inclusion criteria, which required them to be comprised of more than 60% to 80% tumor nuclei and less than 20% to 30% necrotic tissue. Thus, we defined the lymphocyte proportions calculated from the bulk tumor to be intratumoral TILs (TCGA criteria can be found at https://www.cancer.gov/ccg/research/genome-sequencing/tcga). For GSE25066 and GSE20271, gene expression data in the indicated accession, GPL format, were obtained from the Gene Expression Omnibus (GEO) database (https://www.ncbi.nlm.nih.gov/geo/) using the GEOquery package in *R*, and gene symbols were mapped from probe IDs based on the annotation information of the corresponding GPL platform. For GSE96058, GSE163882, and GSE123845, which were RNA sequencing data, we used the normalized data provided by the authors. All data were downloaded in July 2022. Institutional Review Board approval was waived since all the cohorts used are de-identified and obtained from the public domain.

### Estimation of Intratumoral TILs From Transcriptome Analyses

To estimate the fraction of intratumoral TILs in bulk tumors, we employed several deconvolution tools to estimate cell fractions from transcriptomics in both the TCGA and GSE96058 cohorts. Deconvolution tools estimate cell fractions from transcriptomics data by comparing the expression levels of cell-specific marker genes to reference gene expression profiles for each cell type. These tools use mathematical algorithms to determine the proportions of different cell types present in a mixed population of cells based on their gene expression signatures. We uploaded gene expression data to the online tool of xCell (https://xcell.ucsf.edu/),^23^ QUANTISEQ (https://icbi.i-med.ac.at/software/quantiseq/doc/),^24^ EPIC (https://epic.gfellerlab.org/),^25^ and CIBERSORT (https://cibersortx.stanford.edu/index.php)^26^ and calculated the cell types included in each tool. Based on these results, we decided to use the whole lymphocyte population estimated by xCell as the computationally estimated TIL score in this study, which includes the cells as shown in Table 1. Definitions of whole T-cells and lymphocyte populations estimated by each platform can be found in Supplementary Table 1, Supplemental Digital Content 1, http://links.lww.com/SLA/E669. For the estimation of cell fractionation in neoadjuvant cohorts, we converted gene expression data into gene symbols and uploaded the normalized expression data to the xCell web tool for the deconvolution of bulk tumors.

**TABLE 1:** The cell types included in the TIL score (whole lymphocytes detected by xCell algorithm)

CD4+ T cells, CD4+ memory T cells, CD4+ naive T cells, CD4+ Tcm, CCD4+ Tem, CD8+ T cells, CD8+ naive T-cells, CD8+ Tem, CD8+ Tcm, Tregs, Th1 cells, Th2 cells, B-cells, pro B-cells, naive B-cells, Memory B-cells, Class-switched memory B-cells, NK cells, NKT

### Evaluation of Immune Function of TCGA and Gene Set Enrichment Analysis

Gene Set Enrichment Analysis (GSEA)^27^ was conducted to evaluate the biological function of TCGA and GSE96058 cohorts using the Hallmark gene sets from the Molecular Signatures Database.^28^ We chose to use only the Hallmark gene sets for GSEA analysis, as they provide a comprehensive representation of the tumor microenvironment. Significant differences were defined as false-discovery rate (FDR) *q*-values below 25%, and the strength of the correlation with the gene set was assessed using the normalized enrichment score (NES). The immune activity level was determined using the cytolytic activity score (CYT), which was calculated based on the expression of granzyme A (*GZMA*) and perforin (*PRF1*) genes.^29^ For calculations of intratumoral heterogeneity, homologous recombination deficiency (HRD), silent and nonsilent mutation rates, and single nucleotide variation and indel neoantigen scores, we utilized the data published by Thorsson et al for the TCGA cohort.^30^ Briefly, intratumor heterogeneity was defined as the subclonal genomic fraction, which was analyzed using the ABSOLUTE algorithm based on copy number variation and mutation information of the tumor. HRD score was defined as the sum of 3 genomic scores: large-scale state transitions, subtelomeric regions with allelic imbalance, and large non-arm level regions with loss of heterozygosity. Mutation rates were calculated using a standardized, normalized, batch, and platform-corrected data matrix and mutation data generated by the PanCancer Atlas Consortium (https://gdc.cancer.gov/about-data/publications/pancanatlas).

### Statistical Analysis

Statistical analyses were conducted using *R* 4.2.1 and various packages, including Biobase 2.58.0, GEOquery 2.60.0, ggplot2 3.3.6, ggpubr 0.4.0, grayzoneSurv 1.0, gtsummary 1.6.2, MatrixGenerics 1.10.0, RcmdrMisc 2.7-1, SummarizedExperiment 1.28.0, survival 3.2-1, survAUC 1.0-5, pROC 1.18.0, S4Vectors 0.30.0, and tidyverse 1.3.1. To investigate the differences in the clinicopathological and biological features between low and high TIL infiltration fraction, patients were divided into low and high TIL groups by the median value within each cohort. The median values for each breast cancer subtype were used, resulting in different median values for each subtype and creating separate low and high groups within each subtype. Comparisons between the 2 groups were conducted using the Kruskal-Wallis and Wilcoxon signed-rank tests. Survival analyses were performed using the log-rank test. *P*<0.05 indicated statistical significance.

## RESULTS

### Establishment of the Tumor Infiltrating Lymphocyte (TIL) Score to Estimate the Number of Lymphocytes in a Sample

**FIGURE 1:**
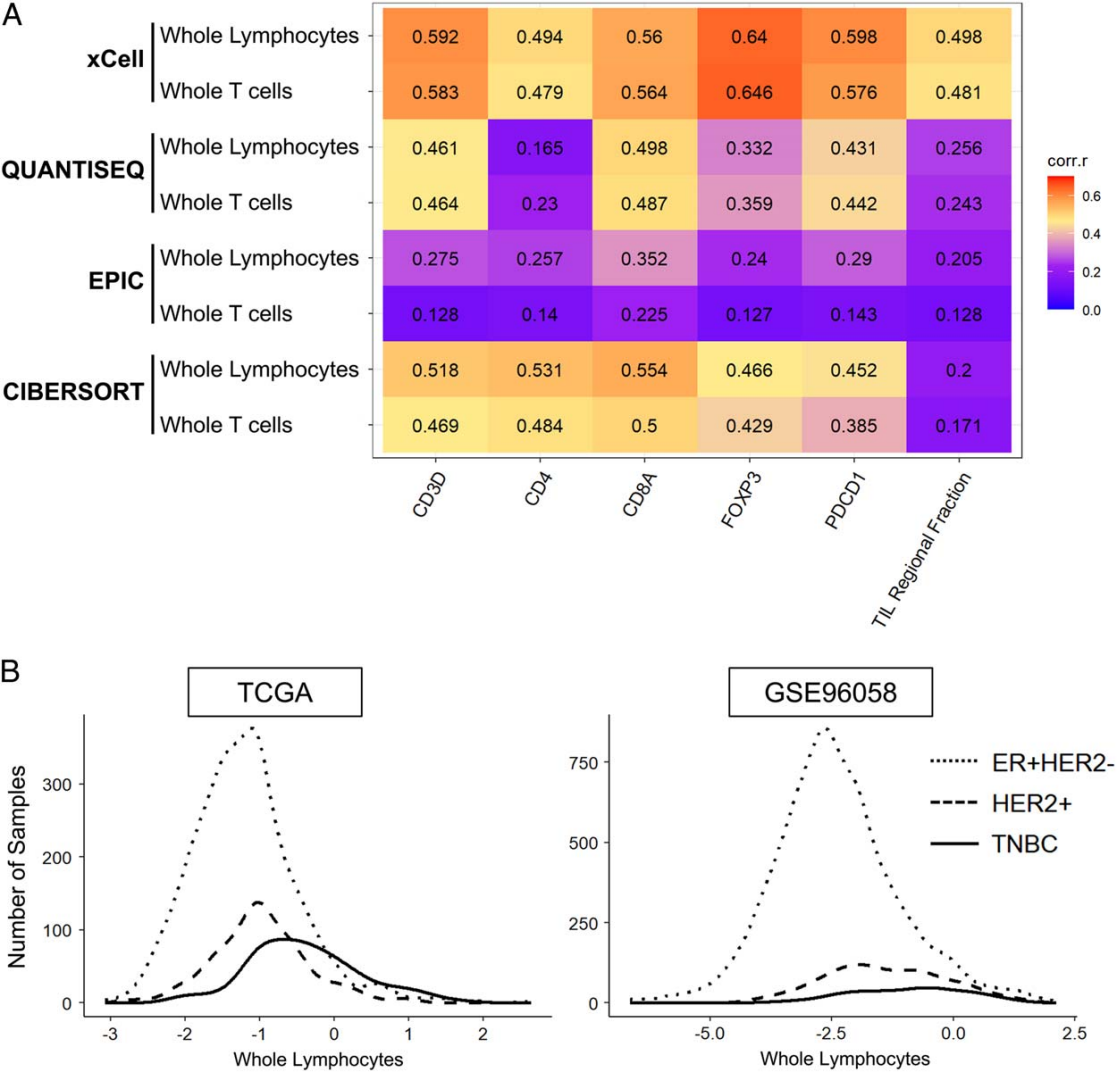
Correlation of whole lymphocytes estimated by the xCell algorithm with TIL-related parameters. A, Correlation Matrix with Spearman’s rank correlation coefficients (corr.r) between lymphocyte markers (*CD3D, CD4, CD8A, FOXP3, and PDCD1*) and pathologically assessed TIL regional fraction, and whole lymphocytes and whole T-cells estimated by multiple deconvolution algorithms. Higher correlation coefficients are shown in red and orange and lower in blue and purple. B, Histograms of intertumoral TILs in each subtype of TCGA and GSE96058 cohorts. The log2 transformation of the sum of whole lymphocytes estimated by the xCell algorithm represents the intertumoral TILs. TCGA indicates The Cancer Genome Atlas; TILs tumor infiltrating lymphocytes.

To accurately quantify the small number of intratumoral TILs, we established a measure to objectively quantify TILs from the transcriptome of a bulk tumor. The correlation between the deconvolution algorithms xCell, QUANTISEQ, EPIC, and CIBERSORT used to estimate the whole T cell population or whole lymphocyte population in the bulk tumor, and parameters that estimated TILs, were measured in the TCGA cohort (Fig. 1A). Gene expressions of CD3D, CD4, CD8A, and FOXP3 were chosen since they are surface markers of different lymphocytes and T-cells commonly used in flow cytometry. PCD1 is a representative marker T cell function, and the TIL Regional Fraction is the stromal TIL infiltration that was evaluated pathologically. We defined the whole T cells and whole lymphocytes by each of the algorithms as shown in Supplementary Table 1, Supplemental Digital Content 1, http://links.lww.com/SLA/E669. The strongest correlations across the board were observed in the whole lymphocytes using the xCell algorithm (xCell Whole Lymphocytes in Fig. 1A).

Based on these results, we decided to use the whole lymphocyte population estimated by xCell as the computationally estimated TIL score in this study, which includes the cells as shown in Table 1. We decided to use this TIL score as a surrogate for intratumoral TILs assuming that the original samples were predominantly collected from cancer-cell-rich areas of the bulk tumors. Figure 1B displays histograms of intratumoral TILs in the TCGA and GSE96058 cohorts, which both show roughly bell-shaped curves. In both cohorts, the median total lymphocyte count is highest in triple-negative breast cancer (TNBC), followed by the HER2-positive subtype, and lowest in the ER-positive/HER2-negative subtype (Fig. 1B).

### Immune-Related Gene Sets are Uniformly Enriched in Tumors With High Intratumoral TILs Regardless of the Subtypes, and the Highest Cytolytic Activity Was Observed in TNBC With High Intratumoral TILs

**FIGURE 2:**
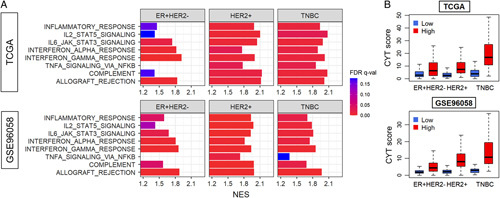
The association of the intratumoral TILs with immune-related gene sets and cytolytic activity in breast cancer. A, Gene set enrichment analysis (GSEA) comparing high versus low TIL groups by subtypes: ER-positive/HER2-negative, HER2-positive, and TNBC in the TCGA and GSE96058 cohorts. Hallmark immune-related gene sets assessed: inflammatory response, IL2/STAT5 signaling, IL6/JAK/STAT3 signaling, interferon-alpha and -gamma response, TNF-alpha signaling through NFκB, complement, and allograft rejection. The x-axis shows NES. Bar color represents FDR. Lower FDR is shown in red and higher in blue, and FDR lower than 0.25 is considered significant. Only pathways that enriched to the high TILs group (ones with positive NES values) are shown, as none of the immune-related pathways were significantly enriched to the low TILs group. (B) Boxplots of cytolytic activity (CYT) score by low and high TIL groups in each subtype in both cohorts. The numbers of high and low groups in each subtype were as follows: ER+HER2-: 292 each, HER2+: 91 and 90, TNBC: 80 and 79, for TCGA, ER+HER2-: 1139 and 1138, HER2+: 196 each, TNBC: 78 and 77, for GSE960598, respectively. The comparisons are performed by Wilcoxon signed-rank test. The line in the box shows the median and the top and bottom of the box show the 25th and 75th percentiles, respectively. FDR indicates false-discovery rate; NES, normalized enrichment score; TCGA, The Cancer Genome Atlas; TNBC, triple-negative breast cancer.

Since TILs include activated immune cells, it is expected that the immune activity of a tumor increases with the number of intratumoral TILs. Gene set enrichment analysis (GSEA) using the Hallmark collection was conducted to assess immune-related pathway activations in intratumoral TIL-high tumors. We divided each cohort into low versus high TILs groups by the median. As expected, TIL-high tumors had multiple significantly enriched immune-related gene sets, including the inflammatory response, IL2/STAT5 signaling, IL6/JAK/STAT3 signaling, interferon-alpha response, interferon-gamma response, TNF-alpha signaling through NFκB, complement, and allograft rejection gene sets in both cohorts, particularly in the TNBC and HER2-positive subtypes (Fig. 2A). None of the immune-related pathways were significantly enriched to the low TILs group. Cytolytic activity (CYT score), was also higher in the TIL-high group compared to the TIL-low group across all subtypes (Fig. 2B). Among the TIL-high tumors, the CYT score was the highest in TNBC, followed by HER2-positive, and the lowest for the ER-positive/HER2-negative subtype. To this end, we demonstrated that intratumoral TIL-high tumors were associated with host immunity activity using computational algorithms.

### Dendritic Cell, Macrophage, Mast Cells, and Monocytes Populations Were Infiltrated in Intratumoral TIL-High Tumors

**FIGURE 3:**
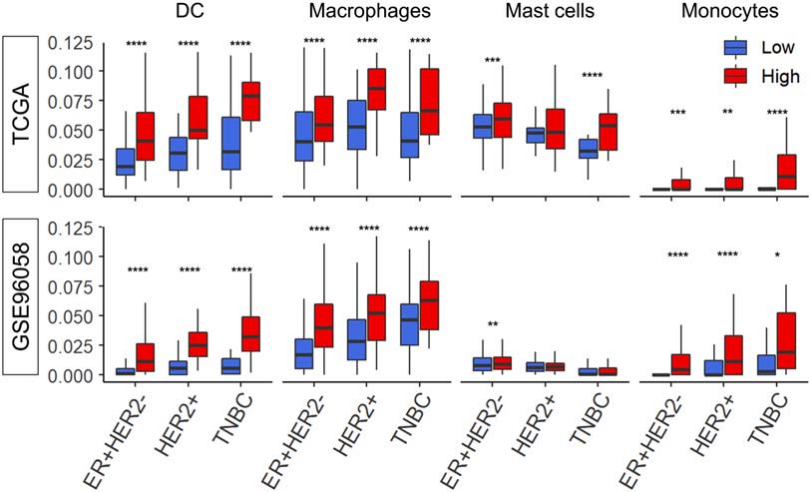
The differentiation of the infiltration fraction of several immune cells in the tumor microenvironment between low and high TIL groups in each breast cancer subtype. Boxplots of dendritic cells (DC), macrophages, mast cells, and monocytes by low and high TIL groups within each subtype; ER-positive/HER2-negative, HER2-positive, and TNBC in the TCGA and GSE96058 cohorts. Red boxes represent TIL-high tumors and blue boxes represent TIL-low tumors, dichotomized by the median. The comparisons were performed using the Wilcoxon signed-rank test. The line in the box shows the median and the top and bottom of the box show the 25th and 75th percentiles, respectively. Results of Wilcoxon signed-rank test *P*<0.05 is taken as significant and shown as follows; ****P<=0.0001, ***P<=0.001, **P<=0.01, *P<=0.05. DC indicates dendritic cells; TCGA, The Cancer Genome Atlas.

Given that immune cells other than lymphocytes also play important roles in the tumor microenvironment, it was of interest whether there is a difference in the infiltration of other immune cells between TIL-low and TIL-high tumors. Using the xCell algorithm, we found that dendritic cells, macrophages, and monocytes were highly infiltrated in the TIL-high tumors regardless of the subtype, while mast cells were highly infiltrated only in the ER-positive/HER2-negative subtype consistently in 2 cohorts (Fig. 3). In addition, we also investigated which lymphocytes infiltrated in the low and high TILs tumors by the subtypes (Supplementary Figure 1, Supplemental Digital Content 1, http://links.lww.com/SLA/E669).

### Intratumoral TIL-High Tumors Were Significantly Associated With Higher Mutations Only in the ER-Positive/HER2-Negative Subtype

**FIGURE 4:**
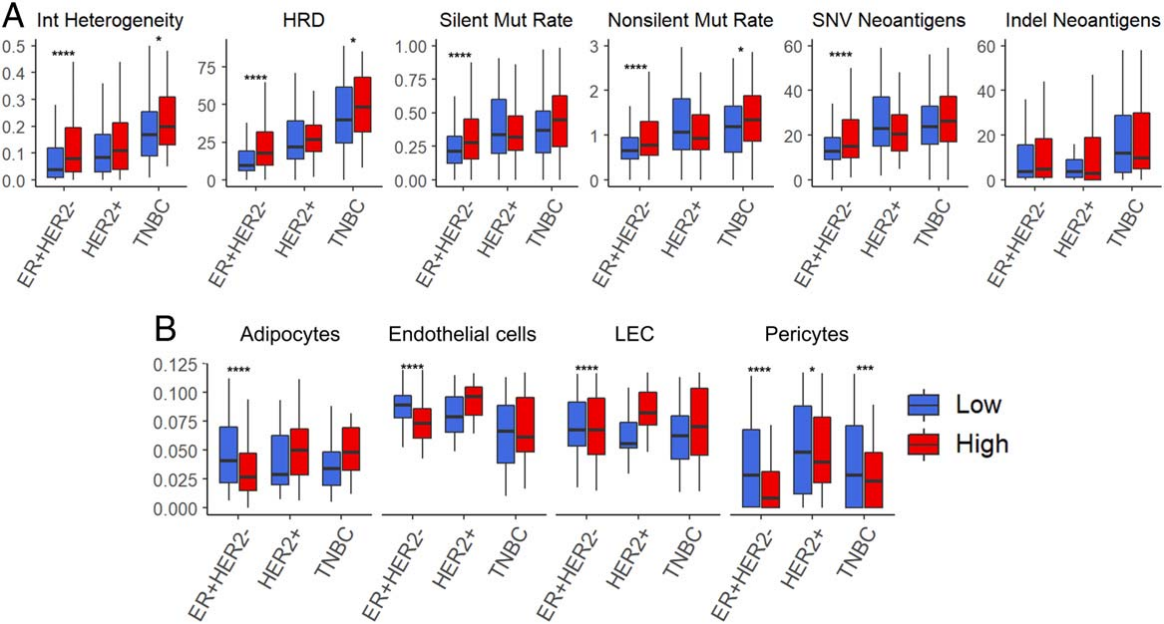
The association of intratumoral TILs with mutations and infiltrating stromal cells in the tumor microenvironment by each breast cancer subtype. A, Boxplots of Intratumor heterogeneity, homologous recombination deficiency (HRD), silent and nonsilent mutation rate, and single nucleotide variation (SNV) and indel neoantigen, precalculated by Thorsson et al^30^ B, The estimated number of stromal cells (adipocytes, endothelial cells, lymphatic endothelial cells (LEC), and pericytes) by low and high TIL groups within each subtype (ER-positive/HER2-negative, HER2-positive, and TNBC) in the TCGA cohorts, calculated by the xCell algorithm. The numbers of high and low groups in each subtype were as follows: ER+HER2-: 292 each, HER2+: 91 and 90, TNBC: 80 and 79, for TCGA, ER+HER2-: 1139 and 1138, HER2+: 196 each, TNBC: 78 and 77, for GSE960598, respectively. The comparisons were performed using the Wilcoxon signed-rank test. The line in the box shows the median and the top and bottom of the box show the 25th and 75th percentiles, respectively. Results of Wilcoxon signed-rank test *P*<0.05 is taken as significant and shown as follows; ****P<=0.0001, ***P<=0.001, **P<=0.01, *P<=0.05. LEC, lymphatic endothelial cells; SNV, single nucleotide variation; TCGA, The Cancer Genome Atlas; TILs tumor infiltrating lymphocytes; TNBC, triple-negative breast cancer.

Since cancers with high mutation rates and neoantigens are known to elicit immunogenicity and cancer immunity,^31^ it was of interest whether intratumoral TIL-high tumors were associated with increased mutation rates and neoantigens. Unexpectedly, intratumor genomic heterogeneity, HRD, silent and nonsilent mutation rates, and single nucleotide variations and Indel neoantigens were all significantly higher only in TIL-high tumors in the ER-positive/HER2-negative subtype (Fig. 4A). In addition, we have previously reported that breast cancers with enhanced angiogenesis,^7^ infiltration of lymphatic endothelial cells,^8^ or adipocytes^32^ were associated with elevated immune response. To this end, we investigated the relationship between intratumoral TILs and stromal cell infiltrations. We found that adipocytes, endothelial cells, lymphatic endothelial cells, and pericytes were significantly less infiltrated in TIL-high ER-positive/HER2-negative subtype tumors, while there was no difference in any of the stromal cells in the other subtypes except for pericytes in the TCGA cohort (Fig. 4B). These results indicate that intratumoral TIL infiltration was associated with higher mutation rates and mitigated stromal cell infiltration, particularly in ER-positive/HER2-negative subtype tumors.

### Intratumoral TIL-High Tumors Most Strongly Correlated With Increased Cell Proliferation in ER-Positive/HER2-Negative Breast Cancer

**FIGURE 5:**
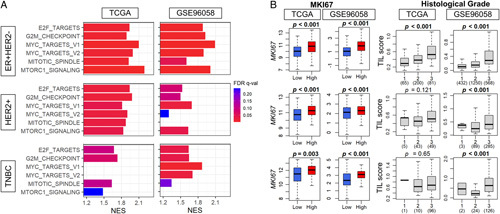
The association of the intratumoral TILs and cell proliferation-related GSEA, Nottingham histological grade, and Ki67 gene expression in breast cancer. A, Gene set enrichment analysis (GSEA) of the Hallmark cell proliferation-related gene sets (E2F Targets, G2M Checkpoint, Myc Targets v1 and v2, Mitotic Spindle, and mTORC1 Signaling), comparing high and low TIL groups in ER-positive/HER2-negative, HER2-positive, and TNBC subtypes in the TCGA and GSE96058 cohorts. The x-axis shows the normalized enrichment score (NES). The bar color represents the false-discovery rate (FDR). A lower FDR is shown in red and higher in blue, and FDR lower than 0.25 is considered significant. B, Boxplots of the TIL score by *MKi67* expression and Nottingham histological grade (grade 1-3) by low and high TIL score in each subtype in both cohorts. The comparisons were performed using the Kruskal-Wallis and Wilcoxon signed-rank test. The line in the box shows the median and the top and bottom of the box show the 25th and 75th percentiles, respectively. FDR indicates false-discovery rate; NES, normalized enrichment score; TCGA, The Cancer Genome Atlas; TILs tumor infiltrating lymphocytes; TNBC, triple-negative breast cancer.

Given that TIL-high tumors were significantly associated with high mutation rates among ER-positive/HER2-negative breast cancer that are known to be relatively less mutated compared with the other subtypes, it was of interest to investigate their relationship with cell proliferation. As predicted, we found that TIL-high tumors were enriched in all the Hallmark cell proliferation-related gene sets (E2F Targets, G2M Checkpoint, Myc Targets V1, Myc Targets V2, Mitotic Spindle, and mTORC1 Signaling) (Fig. 5A). This result was most consistently significant in both the TCGA and GSE96058 cohorts for the ER-positive/HER2-negative subtype compared with TIL-low tumors (Fig. 5A). The TIL-high tumors showed significantly higher expression of *MKI67* (the coding gene of Ki67, a proliferation marker) in all the subtypes consistently in both cohorts (Fig. 5B). Further, TIL-high tumors were significantly associated with higher Nottingham histological grade, which is the morphological assessment of cancer cell proliferation, in the ER-positive/HER2-negative subtype in both cohorts (Supplementary Table 2, Supplemental Digital Content 1, http://links.lww.com/SLA/E669). TIL score significantly correlated with grade in the ER-positive/HER2-negative subtype in both cohorts but only in GSE96058 in the other subtypes (Fig. 5B). This may be due to the very small number of Grade 1 patients in the HER2-positive and TNBC subtypes in TCGA.

Intratumoral TIL-high tumors were not always associated with pathologic complete response (pCR) after neoadjuvant chemotherapy but were consistently associated with better overall survival in the HER2-positive and TNBC subtypes.

**FIGURE 6:**
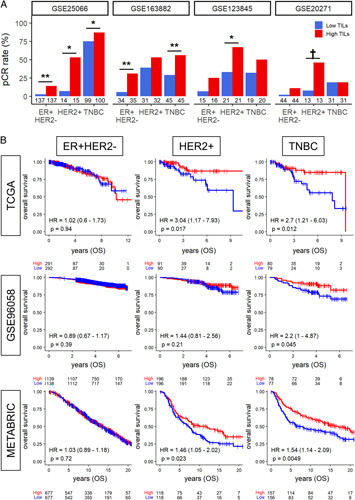
Relationship between pCR rate after neoadjuvant chemotherapy and TILs score. (A) Bar plots show the pCR rate after neoadjuvant chemotherapy among the TIL-high group (red) and the low TIL-low group (blue) in early-stage breast cancer within each cohort. The numbers located below each bar indicate the number of samples analyzed within the respective groups. Results of Fisher exact test *P*<0.05 is taken as significant and shown as follows; **P<0.01, *P<0.05, †*P*<0.1. (B), Estimated survival (Kaplan-Meier) with log-rank and hazard ratio (HR) of overall survival (OS) for breast cancer patients with low and high TIL score groups within each breast cancer subtype in the TCGA and GSE96058 cohorts. OS indicates overall survival; pCR, pathological complete response; TCGA, The Cancer Genome Atlas; TILs tumor infiltrating lymphocytes; TNBC, triple-negative breast cancer.

The abundance of pathologically determined stromal TILs is known to be a surrogate marker of response to neoadjuvant chemotherapy in breast cancer.^33,34^ Therefore, we expected that intratumoral TILs assessed by our TIL score would be associated with complete response to neoadjuvant chemotherapy in the HER2-positive and TNBC subtypes, which is almost always the case in morphologically evaluated stromal TILs. We utilized 4 primary breast cancer cohorts (GSE25066, GSE163882, GSE123845, and GSE20271) that were treated with conventional anthracycline and taxane neoadjuvant chemotherapy and compared the ratio of the patients who achieved pathologic complete response (pCR) by high versus low intratumoral TILs in each subtype (Fig. 6A). We found that pCR rates were uniformly higher in TIL-high tumors; however, only half of the TIL-high tumors showed significantly higher pCR rates compared to TIL-low tumors regardless of the subtype across all cohorts. These results suggest that intratumoral TILs estimated by our score did not always predict the response to neoadjuvant chemotherapy, unlike morphologically assessed stromal TILs, although our observations could be driven by the relatively small numbers of patients in the various subtypes in the cohorts we analyzed.

Lastly, we investigated whether intratumoral TIL score correlated with survival outcome. Overall survival analysis comparing the high versus low TIL group showed no difference in the ER-positive/HER2-negative subtype in any of the cohorts investigated, but TIL-high tumors were associated with significantly better survival in the HER2-positive subtype in the TCGA and METBARIC cohorts and in the TNBC subtype in all 3 cohorts. This result demonstrates that the estimated transcriptomic TILs score aligns with the results by morphologically assessed stromal TILs.

## DISCUSSION

Studies have shown that pathologically evaluated stromal TILs correlate with clinical outcomes.^35–37^ They were scored following the International TILs Working Group guideline by assessing the percentage of TILs in the stroma,^3^which has been shown to be reproducible.^38^ We estimated the number of intratumoral TILs using computational algorithms (TILs score) on transcriptomes of breast cancers that allow for the quantification of very small numbers of cells. Theoretically, intratumoral TILs were expected to have stronger effects on cancer cell biology due to their closer proximity to the tumor cells. We chose the entire sum of all the cell types of lymphocytes estimated by the xCell algorithm as the TIL score among several deconvolution algorithms because of the highest correlation with multiple signals that represent TILs. As expected, intratumoral TILs were more abundant in TNBC compared with ER-positive/HER2-negative breast cancer. Immune response was uniformly enriched in TIL-high tumors regardless of subtype when cohorts were dichotomized into high versus low TIL tumors by the median, and the difference in cytolytic activity by TILs was largest in TNBC. Macrophages, dendritic cells, and monocytes were more common in TIL-high tumors regardless of tumor subtype. Interestingly, TIL-high tumors were significantly associated with higher mutation burden only in the ER-positive/HER2-negative subtype. Moreover, this subtype was most strongly associated with increased cell proliferation in the analysis of biological, pathological, and molecular characteristics. Surprisingly, high intratumoral TIL levels were not always associated with pCR after anthracycline and taxane neoadjuvant chemotherapy. However, they were consistently associated with improved overall survival in the HER2-positive and TNBC subtypes, but not in the ER-positive/HER2-negative subtype.

Among various mechanisms involved in the growth and progression of cancer, the interaction between cancer cells and host immune cells is critical. TILs have drawn considerable interest not only because of immunotherapy development but also because the tumor immune microenvironment has many essential roles in breast cancer progression. Two types of TILs, stromal and intratumoral, exist based on their location. Stromal TILs are the lymphocytes that are scattered or accumulated in the stroma between cancer cells/clusters and do not directly interact with cancer cells.^3^ Most studies evaluated stromal TILs following the guidelines of the International TILs Working Group,^3^ or the European Working Group.^39–41^ Studies have shown that stromal TILs in breast cancer is a predictive biomarker of response to treatment, particularly in TNBC and, less so for HER2-positive breast cancers.^3^ Intratumoral TILs, conversely, are defined as lymphocytes that are in direct contact with cancer cells without intervening stroma. Therefore, theoretically, they are more biologically relevant and influential to cancer cells than stromal TILs. However, intratumoral TILs are difficult to manually evaluate and score using Hematoxylin and Eosin staining, as the lymphocytes can be difficult to differentiate from the other small cells, such as small tumor cells or apoptotic bodies.^4,5^ To overcome this challenge, this study estimated the number of intratumoral TILs by analyzing the gene expressions in bulk tumors using a computational algorithm, the TIL score. We have previously reported the clinical relevance of intratumoral CD8^+^ T-cells^12^ and regulatory T-cells^42^ using the same method. This approach allows us to objectively quantify even a very small number of cells. Several algorithms have been reported to analyze the fraction of each cell type by deconvolution of bulk tumor gene expression. Since each algorithm has its own features, we compared four representative algorithms, including xCell, QUANTISEQ, EPIC, and CIBERSORT, and found that the xCell algorithm best reflects the infiltrating fraction of TILs in the breast cancer tumor microenvironment.

Further, we evaluated the clinical relevance of intratumoral TILs in detail for each subtype, which is critical since TILs may have unique biological features depending on the subtype.^7^ It is well known that TNBC has higher immune cell infiltrations than the other subtypes.^35^ In contrast, Wolf et al have shown that ER-positive breast cancer with high immune scores may also benefit from immune checkpoint inhibitors.^43^ In our study, we found that in all subtypes, the higher the intratumoral TILs, the higher the expressions of multiple immune-related gene sets, and the higher the infiltrations of dendritic cells, macrophages, and monocytes. Conversely, intratumoral TILs were associated with different features by subtypes. Interestingly, in the ER-positive/HER2-negative subtype, which is generally known to have the least immunogenicity and lowest proliferation among all breast cancer subtypes, TIL-high tumors were particularly associated with higher immune response, mutation rates, and cell proliferation compared with TIL-low tumors.

We assessed the cell proliferation by the enrichment of GSEA and expression of the Ki67 gene of the entire bulk tumors, which include both cancer cells and immune cells. We believe that cancer cell proliferation was enhanced in ER-positive/HER2-negative breast cancer because of the following reasons. One, we found that TIL-high tumors were associated with higher Nottingham histological grade, which is a morphological assessment of the proliferation of cancer cells and not the lymphocytes. Two, the majority of the cells in the samples are cancer cells, according to the TCGA. Three, high proliferation was seen in the ER-positive/HER2-negative subtype that is known to have less lymphocytes, and not in the TNBC subtype that is known to have abundant lymphocytes. With that said, our data do not exclude the possibility that cell proliferation of TILs can be enhanced as well.

The counterbalance mechanism between enhanced immunity by high TIL infiltration and aggressive cancer biology may have played a role in the no survival impact by intratumoral TILs in ER-positive/HER2-negative breast cancer, similar to the mechanism we have previously reported in different settings.^44^ In addition to lymphocytes, which are key players in the tumor microenvironment, other immune cells, such as dendritic cells and macrophages, may also affect the immune microenvironment. Tumor-associated macrophages (TAMs) have been reported to be associated with tumorigenesis, metastasis, and treatment resistance in breast cancer, but they also have anti-tumor effects as well.^45^ Although only a few studies reported on the relationship between TILs and other immune cells, Kuroda et al showed a correlation between TAMs and TILs and their impact on the prognosis of TNBC. TNBCs with low TILs and high TAMs had worse recurrence-free survival and overall survival than those with high TILs and low TAMs.^46^ In addition, Chen et al found that macrophages were more abundant in all subsets of high TILs tumors, including CD45+, CD3+, CD4+, CD8+, and FOXP3+,^47^ which is consistent with our findings. On the contrary, TIL-high tumors in TNBC were consistently associated with a better prognosis, suggesting that the intratumoral TIL score may be a prognostic marker in TNBC. Although large studies on intratumor TILs and prognosis are still scarce, using more than 2000 prospectively collected samples from a phase III adjuvant randomized breast cancer trial, Loi, et al reported that intratumoral TILs were associated with better disease-free and overall survival in TNBC, whereas they were not in ER-positive/HER2-negative or HER2-positive subtypes.^2^ Furthermore, Denkert et al also reported statistically significant linear relationships between the number of intratumoral TILs and improved outcomes in TNBC, which was also confirmed by Loi et al^1,2^

Neoadjuvant chemotherapy is now a common practice for breast cancer patients, and the association of TILs with pCR after chemotherapy in HER2-positive breast cancer and TNBC is widely known.^33,48,49^ Stromal TILs are usually measured in this context, and few investigations have studied intratumoral TILs. Denkert, et al reported that intratumoral TILs were a significant and independent predictive biomarker for pCR in 2 independent cohorts.^1,50^ Khoury et al also reported that both stromal and intratumoral TILs were independent predictors for pCR in TNBC, whereas only intratumoral TILs significantly predicted pCR in ER-positive/HER2-negative subtype breast cancer.^5^ Neither study showed significance in the HER2-positive subtype. Our study showed significant associations between intratumoral TILs and pCR rates in about half of the cohorts, regardless of subtypes. The small number of patients in some cohorts may be one possible reason for the lack of statistical significance. Our results may also suggest different functions of intratumoral TILs from stromal TILs in the context of predicting therapeutic response. Taken together with the recent popularity of immunotherapy for breast cancer, we believe that future prospective studies to investigate the utility of intratumoral TILs measured by our score as one of the options for patient selection for immunotherapy is warranted.

There are several limitations to this study. First, although we utilized multiple independent large patient cohorts, this is a retrospective investigation with publicly available cohorts of previously published studies. The cohorts we examined vary significantly from each other with respect to patient background, clinical characteristics, and number of patients as well as sample handling, and any imbalance among the cohorts could confound our study. The median was used to divide high versus low TILs in this study; however, different cut-offs may warrant further investigation given that the other pathological studies used them. Furthermore, we could not investigate the association of the TIL score with the effects of treatments other than neoadjuvant chemotherapy, such as adjuvant radiation therapy or immunotherapy, due to the lack of data. We defined the lymphocytes in the bulk tumors to be intratumoral TILs because they were sampled from cancer-cell-rich parts of the tumor. However, we cannot exclude the possibility that some stromal TILs may be included in the sample. Finally, this study did not assess the biological mechanisms that underlie their clinical findings. Further research on the different roles of TIL subclasses in each molecular subtype of breast cancer will help us further understand the precise mechanisms of TILs and provide more evidence for the use of immunotherapy in different molecular subtypes of breast cancer.

## CONCLUSION

Intratumoral TILs estimated by transcriptome analysis are associated with increased immune response and cell proliferation in the ER-positive/HER2-negative breast cancer subtype and better survival in the HER2-positive and TNBC subtypes, but not always with pCR after neoadjuvant chemotherapy.

## Supplementary Material

## DISCUSSANT

### Dr Elizabeth Mittendorf (Boston, MA)

Beth Mittendorf from the Brigham and Women’s Hospital in Boston. I do have a few disclosures, I don’t think relevant to this presentation, but I’ve been on scientific advisory boards for AstraZeneca, BioNTech, Genentech, and Merck.

With the success of immunotherapy in treating multiple different cancer types including breast cancer, there’s obvious and significant interest in better defining immunologic aspects of the tumor microenvironment to determine if there are aspects of that TME that are either prognostic or predictive of response to therapy. TIL have been looked at extensively in breast cancer where TIL data are available on well over 20,000 patients, and there’s been robust associations with better prognosis in triple-negative and HER2-positive breast cancer.

The presence of TIL has also been shown to be predictive of response to neoadjuvant therapy, but I think it’s quite interesting to note that in trials such as GeparNuevo (00:52), which randomized patients to chemotherapy or chemotherapy plus immunotherapy, the presence of TIL simply predicted response to treatment. It didn’t differentiate the response to the immunotherapy. Now in that study as well as others, the TIL assessment has been performed on H&E stained slides as was just described. What the TIL Working Group advises is enumeration of stromal TIL. In their guidelines, they stated that the TIL should be reported for the stromal compartment as it’s more reproducible, and as the author noted, there are fewer intratumoral TIL. These intratumoral TIL are more heterogeneous, and they’re more difficult to observe on H&E slides.

However, as the authors rightly point out, it may be more interesting to know about the intratumoral TIL because those lymphocytes have an interface with the cancer cells so may be predicted or hypothesized to be more cytotoxic against the tumor. So the authors have characterized intratumoral TIL and the association of those TILs with response and survival using a fairly sophisticated computational method.

Now one of the things that you looked at was the association of TIL with other immune cells in the tumor microenvironment. You found that the TIL-high tumors were highly infiltrated with macrophages, dendritic cells, and monocytes, and this may not really be surprising in that those are the antigen-presenting cells.

We know that TIL is a broad term and includes T-cells, B-cells, NK cells, and then within that, there’s further differentiation, CD4, CD8, memory, naïve, exhausted, etc so one limitation of previous studies is that the specific TIL subtype is not enumerated. I would suggest your data gives you the opportunity to look at that, and so that’s my first question to you, have you actually looked at the specific type of TIL that contributed to the infiltrate?

And then my second question is related to the pace at which this field and technology is advancing. There are now multiple studies published that look at TIL on single-cell RNA seq dat. Can you comment on how your results compare to those reports?

And related, my final question, we don’t currently send breast tumors for transcriptomic analysis in the context of clinical practice, so can you comment on how you think the field needs to move with respect to routine pathologic assessment of the tumor to identify patients who may respond to different immunotherapeutic approaches?

Thank you.

### Response from Kazuaki Takabe

Dr. Mittendorf, thank you very much for those constructive and insightful comments. As for your first question, we did look at the specific type of TILs, namely CD8 and regulatory T-cells, in regard to response to treatment and survival.

We published in International Journal of Molecular Sciences in 2020 that despite our expectation, CD8 cell number did not associate with a high ratio of pathologic complete response to neoadjuvant chemotherapy in our assessment, but it did with the survival. On the contrary, we published in Cancers 2020 that breast tumors highly infiltrated with Tregs was significantly associated with higher pathologic complete response rate, but not with survival.

We also had a chance to look into plasmacytoid dendritic cells, and we did not see any association with response to treatment, neither with survival, which we published in Cancers 2020. With that said, as you have mentioned, new cohorts are reported and new algorithms are developed pretty much every month. So these analyses may give us different results when done with updated data.

Single-cell sequencing data allows us to investigate the transcriptome of each cell, which provides higher resolution than that of a bulk tumor, and we used it as well in the other studies. However, single-cell sequencing has a couple of limitations. The sequence depth is not as deep as the bulk tumor at this point, thus expressions of many of the genes end up undetected. The number of cells analyzed from a sample is limited, thus whether the cells that are analyzed represent the whole bulk tumor is unknown. Finally, the cost of measuring a sample by single-cell sequence is $3000, as opposed to mRNA-sequence of a bulk tumor is about $500 in our institution at this point. With that said, technology advances at an unbelievable pace and we are hopeful that these limitations will be gone in no time.

In regard to how I think about how to identify patients who may respond to different immunotherapeutic approaches. Given the research I do, I am a big advocate in obtaining the most comprehensive and robust data possible on whatever samples that we can lay our hands on. The strength of the pathological assessment is that it assesses the morphology but the limitation is the number of stainings possible. Thus I foresee a future in spacial transcriptomics that captures the morphology with robust data. Spatial transcriptomics technology that allow us to utilize the fixed samples to run transcriptome is expected to overcome our current biggest barrier, at least in our institution, which is the competition with the pathologists on the samples who want to secure and fix them for the clinical assessment.

But in the future, is the transcriptome the answer? I do not think so. We are using transcriptome because it’s comprehensive. We can see 22,000 gene expression profiles. However, clearly gene expression is not the end product, protein is. The reason we are not into the proteomics is because it is not comprehensive at this point. To this end, once the technology catches up and when we are able to get the comprehensive mass spec-based proteomics, most likely that will be the direction of the field.

Thank you very much for your time and effort of reading our paper.

### Dr Ronald Weigel (Iowa City, IA)

Dr. Takabe, this is a nice study and I appreciate this kind of basic science work being presented here. I have two questions. ER-positive tumors are often poorly responsive to neoadjuvant chemotherapy, so the first question is are the differences in TILs related to potential differences in ER-positive vs. ER-negative tumors and might that account for poor response of ER-positive tumors to neoadjuvant chemotherapy?

My second question is can you use these data to predict response? Can you predict response to anti-PDL treatment based on the findings of different TIL type of invasion found in tumors?

### Response from Kazuaki Takabe

Thank you very much for these questions, and the short answer to our first question is yes. As you clarified, the ER-positive tumors are less likely; however, there are some patients that do respond to neoadjuvant chemotherapy. Our approach were able to tease out these patients, and that is one of our messages. And we do speculate that less TILs may be one of the reasons why ER-positive tumors are less likely to respond.

### Dr Ronald Weigel (Iowa City, IA)

What about prediction to anti-PDL therapy?

### Response from Kazuaki Takabe

Yes, the current study was unable to investigate the relationship between intratumoral TILs and immunotherapy since we were able to access only a single cohort that underwent immunotherapy. Because we were unable to validate the results, we decided not to include those results in this study. Since the neoadjuvant immunotherapy is becoming more the standard now, we are expecting to have access to more of these cohorts, and I hope that I can report our analysis in the future.

### Dr. Alastair Thompson (Houston, TX)

Alastair Thompson, no disclosures. Thank you so much for that beautiful description of the work you’ve done. I wonder whether it’s now timely to use some of the technologies we actually have. I’m thinking of something like Vectra to look at six or eight markers in situ on formalin-fixed paraffin-embedded tissues before and even after neoadjuvant therapy and therefore be able to select which markers you use and give us some truly meaningful output as to which lymphocytes are there and therefore direct our adjuvant or even our neoadjuvant therapy.

### Response from Kazuaki Takabe

I think that is our future direction, and I think that is a good idea.

### Dr Diana Farmer (Sacramento, CA)

Can I ask one question? In terms of the patients that have high TILs but don’t achieve a PCR, to me that’s the most interesting cohort to look at, so from your analysis, did you tease out that group and start to specifically look at targets among those that don’t achieve a PCR but have a high TIL?

### Response from Kazuaki Takabe

We did not specifically do that analysis, but that may provide us the insight on the dyfunction of TILs that resulted in less response.
