# Supplementary material for: Intratumoral Tumor Infiltrating Lymphocytes (TILs) are Associated With Cell Proliferation and Better Survival But Not Always With Chemotherapy Response in Breast Cancer
Source: Ann Surg. 2023 Jun 15;278(4):587–97. doi: 10.1097/SLA.0000000000005954 (PMC10481934; doi:10.1097/SLA.0000000000005954)
Supplement: Supplementary file 1 [file sla-278-0587-s001.docx]

| **Supplementary Table1. Definitions of whole T cells and lymphocytes estimated with each tool** | | |
| --- | --- | --- |
| Tool Name | Type | definition in each tool |
| xCell | whole lymphocyte | CD4+ T-cells, CD4+ memory T-cells, CD4+ naive T-cells, CD4+ Tcm, CD4+ Tem, CD8+ T-cells, CD8+ naive T-cells, CD8+ Tem, CD8+ Tcm, Tregs, Th1 cells, Th2 cells, B-cells, pro B-cells, naive B-cells, Memory B-cells, Class-switched memory B-cells, NK cells, NKT |
|  | whole T cells | CD4+ T-cells, CD4+ memory T-cells, CD4+ naive T-cells, CD4+ Tcm, CD4+ Tem, CD8+ T-cells, CD8+ naive T-cells, CD8+ Tem, CD8+ Tcm, Tregs, Th1 cells, Th2 cells |
| QUANTISEQ | whole lymphocyte | B cell, NK cell, T cell CD4+ (non-regulatory), T cell CD8+, T cell regulatory (Tregs) |
|  | whole T cells | T cell CD4+ (non-regulatory), T cell CD8+, T cell regulatory (Tregs) |
| EPIC | whole lymphocyte | B cell, T cell CD4+, T cell CD8+, NK cell |
|  | whole T cells | T cell CD4+, T cell CD8+ |
| CIBERSORTx | whole lymphocyte | T cells CD4 memory activated, T cells CD4 memory resting, T cells CD4 naïve, T cells CD8, T cells follicular helper, T cells gamma delta, T cells regulatory, NK cells resting, NK cells activated, B cells naïve, B cells memory |
|  | whole T cells | T cells CD4 memory activated, T cells CD4 memory resting, T cells CD4 naïve, T cells CD8, T cells follicular helper, T cells gamma delta, T cells regulatory |

| **Supplementary Table2. Histological Grade by subtype of TCGA and GSE96058** | | | | | | |
| --- | --- | --- | --- | --- | --- | --- |
|  | **TCGA** | |  | **GSE96058** | |  |
|  | **High** | **Low** | **p-value** | **High** | **Low** | **p-value** |
| **ER+HER2-** | N = 292 | N = 292 | **<0.001** | N = 1,139 | N = 1,138 | **<0.001** |
| 1 | 18 (11%) | 47 (26%) |  | 115 (10%) | 317 (28%) |  |
| 2 | 88 (53%) | 112 (62%) |  | 552 (49%) | 698 (62%) |  |
| 3 | 60 (36%) | 21 (12%) |  | 450 (40%) | 118 (10%) |  |
| NA | 126 | 112 |  | 22 | 5 |  |
| **HER2+** | N = 91 | N = 90 | 0.12 | N = 196 | N = 196 | **0.002** |
| 1 | 3 (5.8%) | 2 (4.4%) |  | 2 (1.1%) | 1 (0.5%) |  |
| 2 | 18 (35%) | 25 (56%) |  | 31 (17%) | 58 (31%) |  |
| 3 | 31 (60%) | 18 (40%) |  | 154 (82%) | 131 (69%) |  |
| NA | 39 | 45 |  | 9 | 6 |  |
| **TNBC** | N = 80 | N = 79 | 0.26 | N = 78 | N = 77 | **<0.001** |
| 1 | 1 (2.0%) | 0 (0%) |  | 1 (1.3%) | 1 (1.3%) |  |
| 2 | 3 (5.9%) | 7 (12%) |  | 3 (3.9%) | 21 (28%) |  |
| 3 | 47 (92%) | 49 (88%) |  | 72 (95%) | 54 (71%) |  |
| NA | 29 | 23 |  | 2 | 1 |  |
| *p-values are by Pearson's Chi-squared test or Fisher's exact test | | | | | | |


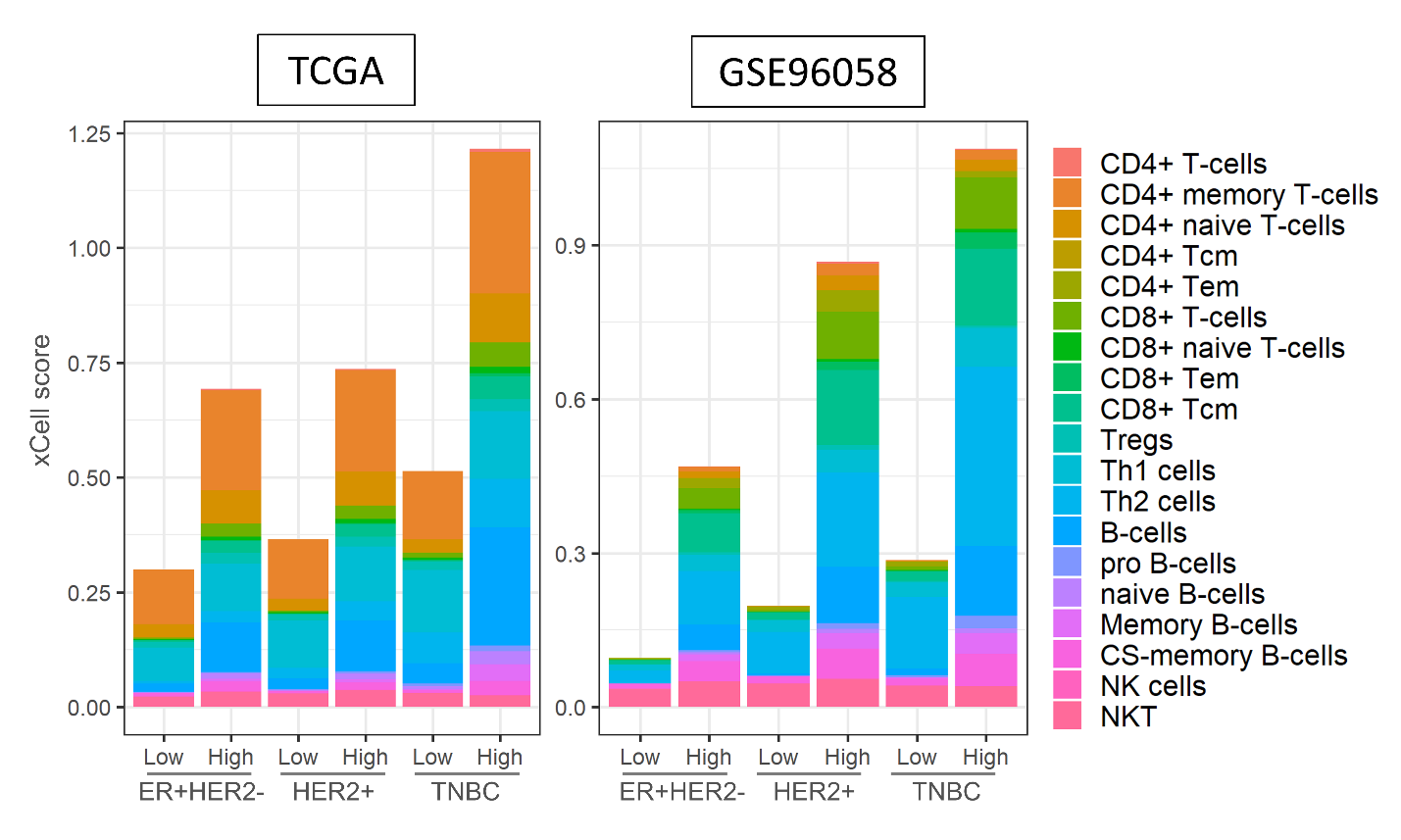


**Supplementary Figure 1. Proportion of Cell Types within Whole Lymphocytes in TCGA and GSE96058 Cohorts**. The bar plot compares the xCell scores of each cell type within whole lymphocytes of high and low TIL groups by ER+HER2-, HER2+, and TNBC subtypes in the TCGA and GSE96058 cohorts. The legend on the right side of the plot indicates the corresponding cell types represented by each color.
